# Improving Security for SCADA Sensor Networks with Reputation Systems and Self-Organizing Maps

**DOI:** 10.3390/s91109380

**Published:** 2009-11-20

**Authors:** José M. Moya, Álvaro Araujo, Zorana Banković, Juan-Mariano de Goyeneche, Juan Carlos Vallejo, Pedro Malagón, Daniel Villanueva, David Fraga, Elena Romero, Javier Blesa

**Affiliations:** Department Ingeniería Electrónica, ETSI Telecomunicación, Universidad Politécnica de Madrid, Av. Complutense, 30, 28040 Madrid, Spain; E-Mails: araujo@die.upm.es (A.A.); zorana@die.upm.es (Z.B.); goyeneche@die.upm.es (J.M.G.); jcvallejo@die.upm.es (J.C.V.); malagon@die.upm.es (P.M.); danielvg@die.upm.es (D.V.); dfraga@die.upm.es (D.F.); elena@die.upm.es (E.R.); jblesa@die.upm.es (J.B.)

**Keywords:** SCADA control system, cyber security, critical infrastructure, reputation system, countermeasure, security framework

## Abstract

The reliable operation of modern infrastructures depends on computerized systems and Supervisory Control and Data Acquisition (SCADA) systems, which are also based on the data obtained from sensor networks. The inherent limitations of the sensor devices make them extremely vulnerable to cyberwarfare/cyberterrorism attacks. In this paper, we propose a reputation system enhanced with distributed agents, based on unsupervised learning algorithms (self-organizing maps), in order to achieve fault tolerance and enhanced resistance to previously unknown attacks. This approach has been extensively simulated and compared with previous proposals.

## 1. Introduction

Security concerns are key issues when developing applications based on sensor networks to monitor and control critical infrastructures, such as water treatment and distribution, oil and gas pipelines, electrical power transmission and distribution, or large communication systems. The use of current SCADA (Supervisory Control And Data Acquisition) systems has already come into question as they are increasingly seen as extremely vulnerable to cyberwarfare/cyberterrorism attacks [1, 2].

In particular, security researchers are concerned about: (1) the lack of concern about security and authentication in the design, deployment and operation of existing SCADA networks; (2) the mistaken belief that SCADA systems have the benefit of security through obscurity through the use of specialized protocols and proprietary interfaces; (3) the mistaken belief that SCADA networks are secure because they are purportedly physically secured; and (4) the mistaken belief that SCADA networks are secure because they are supposedly disconnected from the Internet.

The security of these SCADA systems is important because compromise or destruction of these systems would impact multiple areas of society. For example, a blackout caused by a compromised electrical SCADA system would cause financial losses to all the customers that received electricity from that source.

There are two distinct threats to a modern SCADA system. The first is the threat of unauthorized access to the control software, be it human access or changes induced intentionally or accidentally by virus infections and other software threats residing on the control host machine. The second is the threat of packet access to the network segments hosting SCADA devices. In many cases, there is rudimentary or no security on the actual packet control protocol, so anyone who can send packets to the SCADA device can control it. In many cases SCADA users assume that a VPN is of sufficient protection and are unaware that physical access to SCADA-related network jacks and switches provides the ability to totally bypass all security on the control software and fully control those SCADA networks. These kinds of physical access attacks bypass firewall and VPN security and are best addressed by endpoint-to-endpoint authentication and authorization commonly provided in the non-SCADA world by in-device SSL or other cryptographic techniques. But encryption is not enough for sensor devices because resource restrictions would prevent strong encryption schemes, and low-cost side-channel attacks [3] would still easily reveal the private keys.

The increased interest in SCADA vulnerabilities has resulted in vulnerability researchers discovering vulnerabilities in commercial SCADA software and more general offensive SCADA techniques presented to the general security community [4, 5]. In electric and gas utility SCADA systems, the vulnerability of the large installed base of wired and wireless serial communications links is addressed in some cases by applying bump-in-the-wire devices that employ authentication and Advanced Encryption Standard encryption rather than replacing all existing nodes.

But most of the risks come from the limitations of the sensor nodes: (1) many nodes in the network have very limited resources; (2) pervasiveness implies that some nodes will be in non-controlled areas and are accessible to potential intruders; (3) all these sensor nodes and controlling computers are globally interconnected, allowing attacks to be propagated step by step from the more resource-constrained devices to the more secure servers with lots of private data.

Ciphers and countermeasures often imply a need for more resources (more computation requirements, more power consumption, specific integrated circuits with careful physical design, etc.), but usually this is not affordable for this kind of applications. Even if we impose strong requirements for any individual node to be connected to our network, it is virtually impossible to update hardware and software whenever a security flaw is found. The need to consider security as a new dimension during the whole design process of embedded systems has already been stressed [3, 6], and there have been some initial efforts towards design methodologies to support security [7–9], but to the best of our knowledge no attempt has been made to exploit the special characteristics of sensor networks.

Applications built on sensor networks—SCADA systems being no exception—have to live with the fact that privacy and integrity cannot be preserved in every node of the network. This poses restrictions on the information a single node can manage, and also in the way the applications are designed and distributed in the network.

Of course, the inherent insecurity of embedded systems should not prevent us from striving to avoid compromises. We should guarantee that a massive attack can not be fast enough to escape the detection, isolation, and recovery measures. Therefore we should design the nodes as secure as the available resources would allow.

In spite of the disadvantages of sensor networks from the security point of view, they provide one important advantage for fighting against attacks: redundancy. A sensor network usually has a high degree of spatial redundancy (many sensors that should provide coherent data), and temporal redundancy (habits, periodic behaviors, causal dependencies), and both can be used to detect and isolate faulty or compromised nodes in a very effective manner.

In previous work [10], we proposed the use of reputation systems and non-deterministic routing to secure applications based on wireless sensor networks. In this article, focused on sensor networks for SCADA systems, we propose the use of reputation systems enhanced with distributed agents based on unsupervised learning algorithms (specifically, self-organizing maps) in order to achieve more resistance to previously unknown attacks. Separate detectors are further organized in a distributed system using the idea of immune system organization.

In Section 2. we review some of the most relevant previous approaches. Section 3. describes our approach in detail. In Section 4., some experimental data and algorithms description is shown and discussed. Finally, in section 5., we draw some conclusions.

## 2. Related Work

The problem of security in sensor networks has been widely dealt with by researchers. The classic approach to security in these networks consists in adding an authentication system and encrypting the communications. However, in our opinion this approach cannot be considered secure. Almost every node in sensor networks has very limited resource, so the authentication or encryption algorithms that it uses cannot be complex. Another issue to consider is that updating these algorithms is very difficult in case security failures arise. Finally, nodes in these networks are usually within reach of the attacker, so a large number of side channel attacks can be carried out [3, 11] to obtain security keys.

Different techniques propose secure routing protocols [12, 13] or focus on the routing protocol. Buttyán and Hubaux [14, 15] proposed an architecture to stimulate a correct routing behavior. In their solution, nodes receive a per-hop payment in every packet they forward. Nodes store this payment information in an internal counter. As elements get benefits from routing, they understand cooperation as a benefit not only for the entire network, but also for individual nodes. This approach, however, maintains this cooperation information locally, only shared by nodes that interact directly. In our approach, on the contrary, reputation information is transmitted all over the network, so all nodes are warned about misbehaving nodes. Marti *et al.* [16] proposed mitigating routing misbehavior by detecting non-forwarding nodes and rating every path so those nodes are avoided when the routes are recalculated. The resulting behavior is that non-routing nodes are not included in routing paths (as they are not going to cooperate) but they still can ask others to forward their messages. According to Dawkins [17], this scheme detects the misbehavior but it does not isolate it. In our system, bad-behaving or attacker nodes are properly isolated by rating their reputation as low.

Recently various solutions for intrusion detection for sensor networks have been proposed. The existing IDSs for general computer or Ad Hoc networks could not have been deployed for sensor networks' security since these solutions do not assume limited resources of computational units. Thus, additional efforts have to be dedicated to designing special IDSs compatible with the sensor network limitations. Some of the solutions that propose general IDS schemes are given in [18, 19]. However, to the best of our knowledge, all existing solutions are only capable of detecting known attacks or their variations. In order to detect new attacks, they need to be adjusted by human.

Various solutions that deploy machine learning techniques appeared recently [20, 21]. These solutions support the idea that machine learning techniques offer higher level of flexibility and adaptability. However, these techniques consume significant resources. To the best of our knowledge, nobody has proposed any solution for this issue. Moreover, the feature sets they deploy mostly include those features whose values are known to change under the influence of an attacker, or are known to be weak spots. This is their major deficiency, as relying on these features only the known attacks or their variations can be detected.

There are more complex approaches that provide solutions for the different levels of the problem. The CONFIDANT approach, proposed by Buchegger and Le Boudec [22] is a good effort that solves most of the problems mentioned in this section. CONFIDANT is a protocol that sits over the chosen routing protocol and makes misbehavior less attractive for the nodes than proper routing: nodes watch their neighbors for bad behavior, take into account this behavior in a local reputation system and eventually inform their trusted neighbors on misbehaving nodes. Our approach is in some aspects similar to CONFIDANT. For example, it has a reputation system too, but it is not local, but global.

Intuitively, a node *i* should give more weight to the direct observations made by itself than to the evidence obtained from other nodes. Furthermore, the evidence from different nodes should be weighted on the basis of their respective reputations. The beta reputation system [23] and recent implementations for Mica2 motes [24] are based on these observations to provide resistance against badmouthing attacks. A different scheme is proposed in [25], based on the separation between action trust and recommendation trust.

Either because they hold encryption keys or for other reasons, many approaches demand that the nodes be tamper-proof secure [26]. But this need is more difficult to fulfill with every passing day [27–29]. CONFIDANT's authors reason out that their protocol does not need tamper-proof hardware because other nodes' reputation tables are not alterable by the attack. As far as we know, it is not very clear how they maintain the local trusted nodes list or how trust information is updated. It could be difficult to detect a sybil attack [30, 31] or the impersonation of a trusted node by first reading the trusted node list from a tampered node.

In an early design stage we decided not to depend on the hardware being tamper-proof. In fact, it is our assumption that it isn't *and* that the communications between nodes with limited resources are not secure. Our approach compensates those drawbacks by taking advantage of temporal and spatial redundancy.

In general, most of the studied architectures provide security (by just preventing attacks or by simultaneously detecting attacks and providing countermeasures) in the routing protocol at network level. In [10] we proposed a security infrastructure designed for intelligent environments that takes advantage of the environment and uses information from the application layer. The premises of this solution were redundancy and continuous evolution. We maintain this system as a good solution but it can be improved. In this work we have introduced SOM algorithms to make the system faster in terms of adaptation.

## 3. SOM and WSN

Self organizing maps (SOM), also known as Kohonen networks, are an unsupervised type of neural networks [32]. As in neural networks, the basic idea of SOM has origins in certain brain operations, specifically in projection of multidimensional inputs to one-dimensional or two-dimensional neuronal structures on cortex. For example, perception of color depends on three different light receptors (red, green and blue), plus the eyes capture information about the position, size or texture of objects. It has been demonstrated that this multidimensional signal is processed by the planar cortex structure. Further, it has been demonstrated that the areas of the brain responsible for different signals from the body preserve topology, e.g., the area responsible for the signals that come from the arms is close to the area responsible for the signals that come from the hand. These are precisely the basic ideas of SOM that consist in the following:

1. Multidimensional data and their dependencies are presented and captured in a lower dimension SOM network (usually 2D).
2. The proximity of the nodes in SOM reflects similarity of the data mapped to the nodes.

**Figure 1.**
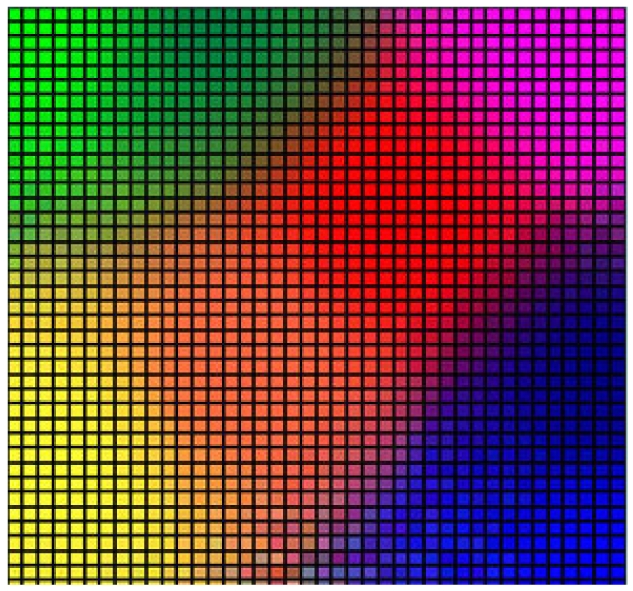
Visualization Property of SOM Clustering.

For these reasons, SOMs have been widely deployed for clustering and good visualization of the problem (Figure 1). They have been successfully deployed in different fields such as image processing [33], robotics [34] (for both visual and motor functions), network security [35], detection of outliers in data [36], etc.

In this work we rely on SOM's ability for detecting outliers [36]. When compared with standard density estimation methods (as kernel density estimator), SOMs are relatively fast and inexpensive when the dimensionality of the data is large. Furthermore, SOMs do not significantly depend on the underlying data model and have no restrictions concerning data dimensionality. This is very important in our case, as we will see in the following text the dimensionality of our data model is variable and grow to be very high.

### 3.1. Underlying Data Model

Bearing in mind that in the case of sensor networks the final objective of an adversary is to compromise the system, and that the easiest way to do it is to send false sensor outputs to the base station in order to give wrong impression of the phenomenon that is being observed, we establish our model of a sensor on sequences of sensor measurements.

Our model is based on two important assumptions:

1. The adversary can capture only a limited number of nodes, which means that most of the output data produced by the sensor nodes is normal. If this is not the case, it means that the adversary can subvert any protocol in the network, which would require for the network re-initialization.
2. Output data produced under the influence of an adversary are statistically different from the output produced during the normal operation of the network. For this reason, we establish the detection of anomalies in data as outlier detection. The definition of outliers is rather fuzzy, but it is considered that an outlier is an observation that lies an “abnormal” distance from other values in a random sample from a population, in other words extreme points in the data cloud.

If any of these assumptions is not fulfilled, our model is not able to work properly.

Our model of sensor outputs relies on sequences of data. In other words, we establish sequential features of data in order to capture the temporal coherence in sensor outputs. In this way, by detecting anomalies in sequences of sensor outputs, we detect the presence of an attacker, including previously unseen attacks, which is the main advantage of our approach. If we had decided to use numerical features, e.g., number of connections between two sensors that occur within a certain period of time, it would have been impossible to detect unknown attacks as we cannot define the numerical features of unknown attacks.

The temporal model proposed here consists of sub-sequences of measurements of a certain size n (called n-grams) and either its frequency or its occurrence in the given sequence (*ϕ*(*x*) in the text). For example, the sequence:

1.00, 1.20, 1.23, 1.22, 1.10, 1.00, 1.20, 1.23

can be characterized with the following model (*n* = 3):

1.001.201.23occurrence 2, frequency 0.331.201.231.22occurrence 1, frequency 0.171.231.221.10occurrence 1, frequency 0.171.221.101.00occurrence 1, frequency 0.171.101.001.20occurrence 1, frequency 0.17

The proposed model offers many advantages. Above all, the time to calculate the similarity between two sequences is linear, which guarantees high speed of the process. Furthermore, it permits the pairwise calculation of similarity, which is essential for its application in anomaly detection based on clustering techniques.

### 3.2. Implemented SOM Algorithm

Our SOM algorithm is implemented in C++ and consists of the following steps [32]:

1. The size of the grid is established, by which the number of the cluster is also known; the centre of each cluster, *i.e.,* grid node, is implemented as a collection whose size is not fixed, *i.e.,* it can be changed on the fly, and whose elements are the *n*-grams defined in the previous text with assigned occurrence or frequency Then, the number of iterations (*N*) is established. Afterwards, each cluster centre is initialized randomly.
2. A random instance from the training data is chosen, *υ*(*t*).
3. The cluster centre closest to the chosen instance is established and named BMU – Best Matching Unit.
4. The BMU radius is calculated according to the Formula 1:
  (1) $$\sigma (t)=\sigma_{0}\text{exp}(-\frac{t}{\lambda }),t=1,2,3\ldots$$
  where *σ*_0_ stands for initial radius, λ is the time constant, and t is the current iteration. At the beginning, *σ*_0_, is quite high, it almost covers the grid, but it decreases during the time (with the time constant λ).
5. Every node, *i.e.,* cluster centre that resides in *ρ*-neighborhood of BMU is adjusted in the following way:
  - If an n-gram of the instance *v(t)* exists in the node, its value (either occurrence of frequency) *ϕ*(*x*) is modified according to the Formula 2:
    (2) ϕ(t+1)=ϕ(t)+Θ(t)L(t)(ϕ(t)−υ(t))
    where *L*(*t*) stands for the learning rate that also decreases during the time according to the Formula 3:
    (3) $$L(t)=L_{0}\exp(-\frac{t}{\lambda }),t=1,2,3\ldots$$
    while Θ(*t*) introduces influence of the distance between a particular node and the BMU and is calculated according to the Formula 4:
    (4) $$\Theta (t)=\exp(-\frac{{\text{dist}}^{2}}{2\sigma^{2}(t)}),t=1,2,3\ldots$$
    where *dist* stands for the distance between the node and the BMU and *ρ*(*t*) is the radius function defined in step 4.
  - If an n-gram of the instance v(t) does not exist in the cluster centre, the n-gram is added to the centre.
6. Steps 2–5 are repeated until all the instances from the training set are chosen.
7. Steps 2–6 are repeated N times.

The principal idea of the algorithm consists in the following: the neighborhood area of the node that corresponds to the current input is adjusted so that all the nodes from the established neighborhood become closer to the input. The change that is introduced in each iteration and the neighborhood that is getting updated are reducing with time, as each node, which at the same time represents a classifier, becomes more stable.

### 3.3. Distance Function

The distance between instances of the presented model is based on kernel functions and is taken from [37]. This is the corresponding pseudo-code:

*function COMPARE*(*X, Y* : *Array*) : *R* *s* ← *e*, *i* ← 1, *j* ← 1 *while*(*i* ≤ |*X*| or *j* ≤ |*Y*|) :  *x* ← *X* [*i*], *y* ← *Y*[*j*]  *if*((*y* = *NIL*)‖(*word*[*x*] = *word*[*y*])) :   *s* ← *s* + *d*(*phi*[*x*], 0)   *i* ← (*i* + 1)  *elseif*((*x* = *NIL*)*or*(*word*[*x*] > *word*[*y*])) :   *s* ← (*s* + *d*(0, *ϕ*[*y*]))   *j* ← (*j* + 1)  *else* :   *s* ← (*s* + *d*(*ϕ*[*x*],*ϕ*[*y*]))   *i* ← (*i* + 1), *j* ← (*j* + 1) *returns*

### 3.4. Outlier Detection

As mentioned before, we treat attacks as data outliers. There are two possible approaches for detecting outliers using SOM algorithm depending on the following two possibilities: detecting outlying nodes or detecting outlying data that belong to non-outlying nodes. For the first case, we calculate the average distance of each node to the rest of the nodes (or its closest neighborhood) (MD). The nodes whose MD values are significantly bigger than the rest are declared to be outlying nodes. In the later case, we calculate quantization error (QE) of each input as the distance from its group centre.

Hence, a node whose average distance is greater than those of the rest of the nodes is considered to be outlying node and all the inputs that belong to it are considered to be anomalies. On the other hand, even if the node to which the current input belongs is not outlying, e.g., outlying data is too sparse for the formation of outlying node(s) to be possible, if its QE value is greater than the rest of the QE values from the same node, *i.e.*, from the medium QE of the node established during the training it is considered to be the proof of the anomaly of the current input.

There are many important advantages that this proposal offers. On one hand, we avoid time consuming and error prone process of collecting only normal data for SOM training if we want to establish a normal behavior and detect attacks as deviations from the established normal behavior. On the other hand, we could train SOM with all the collected data and then label the resulting clusters in a certain way. For example, we can say that the cluster to which the most of the data belongs is the “normal” one, while the rest are intrusive, or measure its *intrusiveness* as the distance from the *normal* one. But, this approach has many flaws, as it can happen that a new intrusion be more similar to the normal data than any of the intrusive ones, so it would falsely be labelled as normal, while our QE measurement is more probable to work in this case.

Every node is being examined by an agent that resides on a node in its vicinity and listens to its communication in a promiscuous manner, where the agent executes the SOM algorithm in order to detect traces of an attack. The system of SOM agents is coupled with a reputation system where each node has its reputation value that basically reflects the level of confidence that others have in it based on its previous behavior. In our proposal, the output of the SOM agent affects on the reputation system in the way that it assigns lower reputation to the nodes where it detects adversarial activities and vice versa. We further advocate coupling the information provided by the reputation system with routing protocol in the way that the nodes with low reputation should not be considered as a routing hop and all the information coming from these nodes should be discarded. In this way, the compromised node remains isolated from the network and has no role in its further functioning. Considering that the attacker that has taken over a node can disable or compromise the SOM agent, we introduce agent redundancy: at least two SOM agents will examine the behavior of each node and both will affect on its reputation.

This approach has many advantages. First of all, SOM algorithm does not use reputation values from the neighborhood, which makes it robust to badmouthing attack, the main problem of reputation systems. Further, it can make the model of data for training and testing on the fly, so it is not necessary to provide the storage for great amounts of data.

### 3.5. Reputation by SOM

Based on the previous definitions of anomaly index, we define the reputation of SOM in the following way:

1. We limit the reputation values to the range [0, 1], where 0 is the lowest possible, meaning that there is no confidence in the node, and 1 the highest possible, meaning the absolute confidence in the node.
2. We define two reputation values, *repQE* and *repMD* based on previously defined *QE* and *MD* values:
  $$\text{repMD}=\frac{\left({\text{maxMD}}_{\text{value}}-\text{anoScMed}\right)}{{\text{maxMD}}_{\text{value}}}$$
  where *maxMD_value_* is the maximum median distance for the current lattice and *anoScMed* is the *MD* value for the best matching unit of the current input. In this way, *repMD* takes values between 0 and 1, where the nodes that are close to the rest (or its proximate vicinity, depending on the definition) have higher reputation and vice versa.

Regarding *QE* value, during the training we calculate the median *QE* for all the nodes in the corresponding SOM lattice. In the testing process, we calculate *QE* value for the corresponding input and calculate *repQE* as the ratio of current *QE* and the median *QE* for its corresponding best matching unit node (or vice versa in order to maintain *repQE* value between 0 and 1). Finally, according to the intuitive reasoning (based on the fact that if the data produced by the presence of an intruder form their own node, it will be significantly distant from the rest; on the other hand, if these data are too sparse and thus not able to form their own group, they will end up belonging to the “normal” nodes, so we have to calculate their *QE* value), we establish the following manner to calculate current reputation:

If (repMD < 0.5) : rep = repMD ;Else : rep = repQE ;

where we take 0.5 as threshold because it is the median value between 0 and 1.

**Figure 2.**
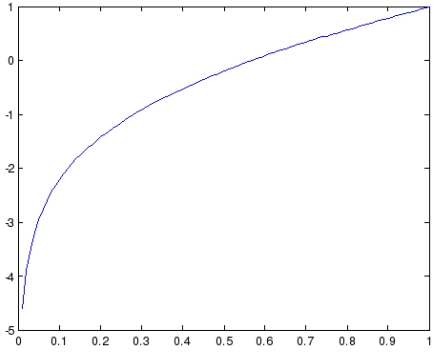
Function for updating reputation values.

Finally, we update the reputation of the node in the following way:

$$\text{cumQE}={\text{rep}}_{(t-1)i}+{\text{rep}}_{ti}+\log(0.99{\text{rep}}_{ti})$$

where *cumQE* stands for cumulative *QE*. If the final value is greater than 1, we truncate it to 1, and in a similar fashion if it is lower than 0, we truncate it to 0. The function *x + log*(0.99*x*) is presented in Figure 2 and provides exactly what we want to achieve: falling of the cumulative reputation if we have small current reputation values and vice versa, and also small changes in the reputation if we are around 0.5. As it can be observed, for the values lower than the 0.3 the values the reputation will fall down quickly, while for the values higher than 0.65 the function rises significantly. Finally, for the values between 0.5 and 0.65 the reputation changes for small amounts.

## 4. Experimental Results

The proposed architecture has been simulated extensively to evaluate its behavior in the presence of attacks of very different nature. Several algorithms have been implemented and used in the reputation server to calculate the reputation of the sensors to make a comparison of its performance: linear algorithm, beta function algorithm and SOM.

In [10] a simple linear algorithm was used to analyse how a reputation system works when integrated in a wireless sensor network. Beta is the most frequent algorithm [23] used in reputation systems and it is also used for WSNs, as shown in [24]. So, we compare both algorithms with a reputation system with SOM algorithms in the context of a SCADA sensor network. We have defined the following characteristics which let us measure the performance and the effectiveness of a concrete algorithm:

- **Detection time**. It is the elapsed time since the attack was started until it is detected, *i.e.*, the reputation of ill-behaved nodes starts decreasing.
- **Isolation time**. It is the elapsed time since the attack is detected until the reputation of every attacker node gets below a threshold. The nodes with reputations below this threshold are not considered for decision making.
- **Isolation capacity**. It is the portion of ill-behaved nodes that are detected as attackers.
- **System degradation.** It is the portion of well-behaved nodes detected as attackers.

### Experiments

One of the most representative identity attacks is the sybil attack, due to its aggressiveness and elusiveness. Besides, it includes most of the identity attacks (thief, mole, clone, etc.) by changing the behavior of the attack.

**Figure 3.**
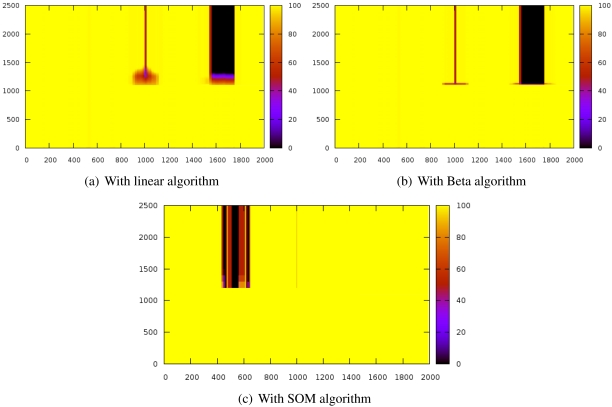
Reputation evolution for a sybil attack.

Figure 3 shows the evolution of the reputation of every node before and after introducing the sybil attack. The Y-axis represents time whereas X-axis indicates space. Z-axis shows reputation values and it is represented by color gradation.

These results have been obtained by simulating a scenario of 2,000 nodes while a sybil node that attacks with 800 identifiers. The sybil node is located in the center of the scenario while the reputation server is located at the origin. The system is working normally until the 1000th iteration, when the sybil attack is launched.

The experiments have revealed that the **detection time** is extremely similar in the three algorithms since the attacks are detected almost immediately.

With the linear algorithms (Figure 3(a)), the reputation of the nodes close to the sybil node is strongly decreased and it takes a while for them to recover the initial reputation. Figure 3(b) shows that this effect is stronger with the beta algorithm but it can restore the reputation of the surrounding nodes of the attacker faster than the linear algorithm. On the other hand, the SOM algorithm (Figure 3(c)) does not affect the neighbors of the sybil node. The three of them also isolate the attacked nodes (thick black stripe), although SOM is more lenient with well-behaved nodes.

It is noteworthy that the SOM algorithm allows very fast confinement of the attack while not affecting so much to the nodes being impersonated (thick dark stripe) and the attacker neighbors (the thin stripe is thinner).

**Figure 4.**
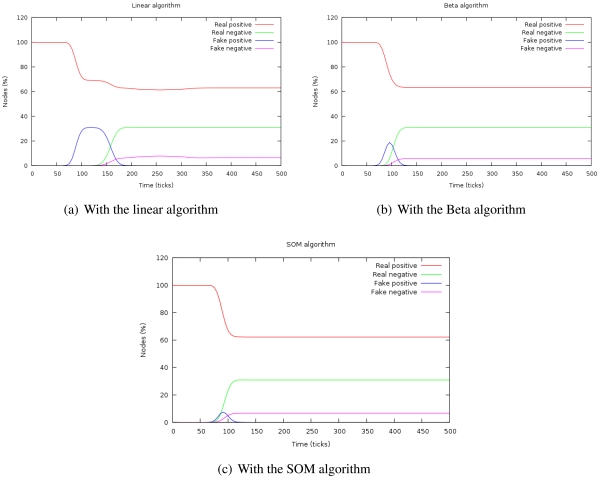
Evolution of the true/false positives/negatives for a sybil attack.

Figure 4 shows the evolution of the percentage of real and false positives and negatives with the three algorithms. When the attack is introduced, the number of false negatives increases during a time. This time is the duration of the attack or **isolation time** and, after that period, it is confined. The height of this curve indicates the depth of the attack in the system.

The **capacity of confinement** can be measured by the number of false positives at the stationary period, that is zero in every case.

**Figure 5.**
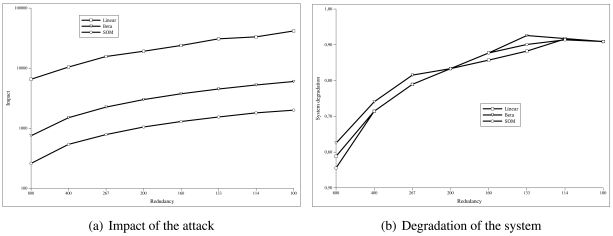
Evolution of the impact of the attack and the based on the sensor node redundancy.

If the area under the curve *number of false positives* is calculated, we can determine the **impact of the attack** because this magnitude takes into account both aspects of the attack: its duration and its depth. Figure 5(a) shows how the impact of the attack changes depending on the redundancy of the sensor nodes. We have simulated the same attack in scenarios in which the number of sensor nodes is decreased. The X-axis represents the number of sensor nodes per each one hundred attacker (*n*). The Y-axis indicates the impact (*I*), calculated as the sum of the false positives given by the Formula 5.

(5) $$I(n)=\sum_{t=0}^{T_{s}}P_{fpt}(n)$$

where *T_s_* is the simulation time and *P_fpt_* is the percentage of false positives at the instant *t*.

Figure 5(b) shows how the system behaves as the redundancy of the sensor network decreases. As we did in the previous simulation, we have used the same attack but decreasing the total number of nodes. The X-axis represents the number of sensor nodes per each one hundred attacker (*n*). The Y-axis indicates the value of the **system degradation** (*D*), given by the Formula 6.

(6) $$D(n)=\frac{N_{{\text{att}}_{T\;s}}}{N_{fpT\;s}\left(n\right)+N_{{\text{att}}_{T\;s}}}$$

where *N_attT s_* is the number of ill-behaved nodes at the end of the simulation (*T_s_*) and *N_fpT s_* is the number of well-behaved nodes considered as ill-behaved ones. The system degradation is optimal when its value is 1.

As we can see in Figure 5(a), the SOM algorithm needs much less redundancy to work compared to the linear or beta algorithms, while obtaining values of system degradation similar to the other algorithms 5(b).

**Figure 6.**
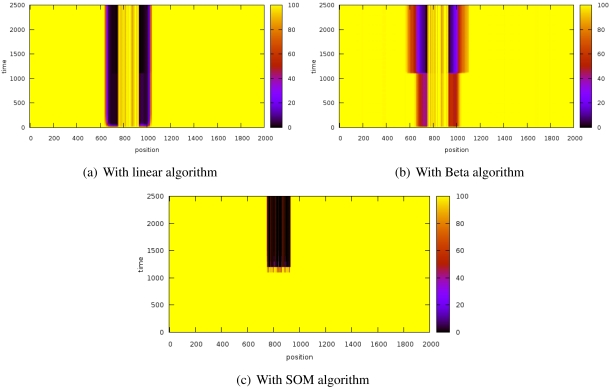
Reputation evolution for a sybil attack after a badmouthing attack.

Since node trusts have no influence in the SOM algorithm, it should be more resistant to reputation attacks like badmouthing. This experiment proves that it is fulfilled. The experiment entails a badmouthing sybil node that attacks the system. The attack decreases the reputation of a group of nodes surrounding the attacker. When their reputation is low enough, the information from sybil identifiers will be used for the decisions. The trust of the neighbors about the attackers will not be taken into account due to their low reputation and, hence, the sybil will not be confined (Figures 6(a) and 6(b)). However, in Figure 6(c) it is shown that the SOM algorithm imperturbably detects and banishes the sybil attack.

The scenario of the experiment has consisted of 180 badmouthing nodes in a small region and, after a while, these nodes become into a sybil node that adopt the identities of the badmouthing nodes.

## 5. Conclusions

Many critical infrastructures are monitored with SCADA systems that process data obtained with a heterogeneous sensor network. SCADA sensor networks are usually composed by many embedded systems with severe resource limitations, joined to the possibility to access physically to the nodes, what make them highly vulnerable to cyberwarfare/cyberterrorism attacks. Cryptography appears as clearly insufficient to maintain data confidentiality and integrity in the network.

We have proposed a holistic solution that assumes this node vulnerability to address security issues in sensor networks, by exploiting redundancy at different levels.

The proposed architecture is based on a reputation system that supports decisions at different levels. It is a trust-based framework where trust data only flows from the sensors to the servers and reputation only from the servers to the sensors. This reputation is also affected by independent agents, using unsupervised learning algorithms, with broader view of the global network. We have demonstrated the effectiveness of this approach with the implementation of anomaly detectors based on self-organizing maps and immune systems.

We have compared the behavior in presence of common attacks with more traditional reputation algorithms, resulting in similar detection time, similar isolation capacity, faster confinement, and highly reduced attack impact even for low redundancy in the sensor network. More importantly, this is done with no previous knowledge about the attacks being performed, and it is more resistant to attack variations, as it is shown in the badmouthing-sybil combined attack.

The resulting approach takes into account practical issues, such as resource limitation, bandwidth optimization and scalability, and it is also well-suited in scenarios with low redundancy. Based on these results we claim that our approach provides a practical solution for developing more secure SCADA applications.
